# Computational modelling of the piglet brain to simulate near-infrared spectroscopy and magnetic resonance spectroscopy data collected during oxygen deprivation

**DOI:** 10.1098/rsif.2011.0766

**Published:** 2012-01-25

**Authors:** Tracy Moroz, Murad Banaji, Nicola J. Robertson, Chris E. Cooper, Ilias Tachtsidis

**Affiliations:** 1 CoMPLEX, University College London, London, UK; 2 Department of Medical Physics and Bioengineering, University College London, London, UK; 3 Institute for Women's Health, University College London, London, UK; 4 Department of Mathematics, University of Portsmouth, Portsmouth, UK; 5 Department of Biological Sciences, University of Essex, Colchester, UK

**Keywords:** NIRS, MRS, mathematical model, neonatal

## Abstract

We describe a computational model to simulate measurements from near-infrared spectroscopy (NIRS) and magnetic resonance spectroscopy (MRS) in the piglet brain. Piglets are often subjected to anoxic, hypoxic and ischaemic insults, as experimental models for human neonates. The model aims to help interpret measurements and increase understanding of physiological processes occurring during such insults. It is an extension of a previous model of circulation and mitochondrial metabolism. This was developed to predict NIRS measurements in the brains of healthy adults i.e. concentration changes of oxyhaemoglobin and deoxyhaemoglobin and redox state changes of cytochrome c oxidase (CCO). We altered and enhanced the model to apply to the anaesthetized piglet brain. It now includes metabolites measured by ^31^P-MRS, namely phosphocreatine, inorganic phosphate and adenosine triphosphate (ATP). It also includes simple descriptions of glycolysis, lactate dynamics and the tricarboxylic acid (TCA) cycle. The model is described, and its simulations compared with existing measurements from piglets during anoxia. The NIRS and MRS measurements are predicted well, although this requires a reduction in blood pressure autoregulation. Predictions of the cerebral metabolic rate of oxygen consumption (CMRO_2_) and lactate concentration, which were not measured, are given. Finally, the model is used to investigate hypotheses regarding changes in CCO redox state during anoxia.

## 1. Introduction

In preclinical neonatology research, piglets are often used as experimental models for human neonates. At birth, their brains have a similar level of maturity to that of human brains [1]. Many studies have used piglets to help understand the effects of oxygen deprivation on cerebral blood flow and metabolism [2,3]. Piglets have also been used to investigate encephalopathy following hypoxia–ischaemia (HI), a major cause of perinatal brain injury [4]. One review found 23 per cent of 292 animal studies of perinatal hypoxic ischaemic encephalopathy had used piglets [5].

The physiological response of the brain during insults such as HI is complex. To help understand it, we have adopted a systems biology approach, and thus developed a mathematical model of circulation and metabolism in the neonatal piglet brain. The model has been created by extending and adapting previous physiological models of the brain [6,7]. Like these, it includes a biophysical sub-model of blood flow on a macroscopic scale, linked to a biochemical sub-model of cellular metabolism. We aim to use the model to help interpret experimental measurements, in particular, non-invasive measurements from near-infrared spectroscopy (NIRS) and magnetic resonance spectroscopy (MRS), both of which have been used extensively in piglet studies.

NIRS uses light to measure concentration changes of oxyhaemoglobin and deoxyhaemoglobin in the blood [8]. Changes in the total haemoglobin concentration in the brain correspond to changes in cerebral blood volume. NIRS has a variety of clinical applications [9], and has frequently been used to study the newborn brain [10].

NIRS can also monitor redox changes at the Cu_A_ centre of cytochrome c oxidase (CCO). The difficulty in interpreting the NIRS Cu_A_ measurement was the main motivation for the development of a previous model by Banaji *et al.* [7], which we will refer to as BrainSignals. We have used this as the basis of our piglet model, since we also aim to investigate interpretation of the Cu_A_ signal. CCO is responsible for oxygen consumption in mitochondrial respiration; therefore, the redox state of Cu_A_ changes with mitochondrial oxygen tension. However, it is also affected by other factors such as adenosine triphosphate (ATP) and adenosine diphosphate (ADP) concentrations.

MRS uses the principle of nuclear magnetic resonance to measure the concentrations of a range of metabolites [11]. Several different nuclei can be measured including ^31^P and ^1^H. ^31^P MRS measures concentrations of the phosphorus-containing metabolites ATP, inorganic phosphate (P_i_) and phosphocreatine (PCr). ADP is not present at high enough concentrations to be measurable. Among the metabolites detectable by proton MRS are lactate and N-acetyl-aspartate.

In this paper, we (i) describe our new development of a piglet brain model, and its addition of simulating the MRS measurable concentrations of lactate, PCr, ATP and ^31^P; (ii) test the model by comparing its simulations of anoxia with experimental results from Springett *et al.* [12] involving simultaneous NIRS and ^31^P MRS measurements; (iii) present hypotheses regarding the interpretation of the reduction in CCO observed during anoxia in these experiments, and the subsequent hyperoxidation during the resuscitation phase; and (iv) predict CMRO_2_ and lactate concentrations, which were not measured, and propose a hypothesis regarding brain autoregulation in 1-day old piglets.

## 2. Methods

**Figure 1.**
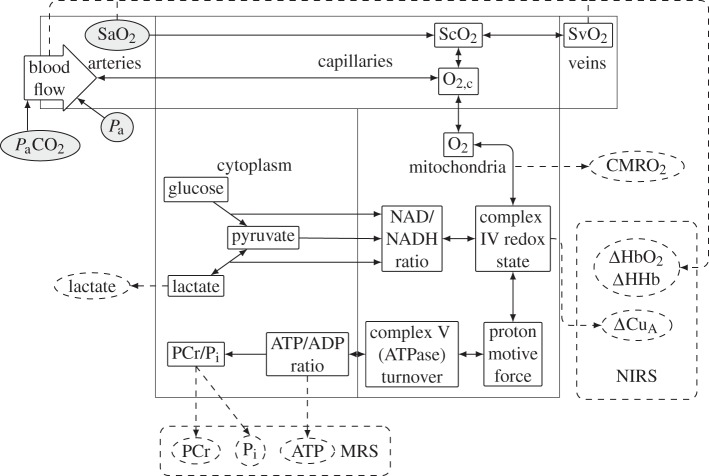
Schematic of the model showing compartments and important variables. Inputs are shown in shaded ovals, outputs in dashed ovals and model variables in rectangles.

A schematic diagram of the model is shown in figure 1. It was based on BrainSignals [7], a model designed to simulate NIRS signals in the healthy adult brain. It was adapted to the neonatal piglet brain by changing the values of appropriate parameters. Of the 107 explicitly set numerical parameters in our model, 78 are taken from BrainSignals, and 11 of their values have been changed as shown in table 1. As well as these parameter changes, the model was expanded from BrainSignals to simulate variables that are measured by MRS. These include the ^31^P MRS measured variables ATP, PCr and P_i_, and the lactate concentration measured by ^1^H MRS.

**Table 1.** Table of parameters from BrainSignals with values changed to represent the anaesthetized piglet brain.

parameter	description	units	BrainSignals	piglet	source
CBF_n_	normal CBF	ml 100 g^−1^ min^−1^	49	46	[13]
[CCO]_tis_	total CCO concentration in tissue	µM	5.5	2.2	[14,15]
Cu_A,frac,n_	normal fraction of CCO oxidized		0.80	0.67	[15]
CMRO_2,n_	normal CMRO_2_	mol 100 g^−1^ min^−1^	155	114	[13,16]
*P*_a_ and *P*_a,n_	mean arterial blood pressure	mmHg	100	50	[13]
[Hbtot] and [Hbtot]_n_	total blood haemoglobin conc	mM (×4)	9.1	5.4	[17]
*V*_blood,n_	normal brain blood fraction		0.0400	0.0325	[16]
*P*_ic_ and *P*_ic,n_	intracranial pressure	mmHg	9.5	4.5	[18]

The approach for this model expansion followed the approach taken in BrainSignals i.e. the model was constructed to represent the underlying physiology. However, processes that are not commonly followed by non-invasive measurements necessarily require simplification to prevent the model from becoming overly complicated. Therefore, some processes and parameters are included which are chosen to fit relevant experimental data. The expansion required the addition of 29 numerical parameters shown in table 2. Where possible, these were set from experimental measurements, but some values were taken from other models. In addition, several rate constants were set by flux balance analysis, subject to the constraints of the explicitly set parameter values. A description of the piglet model is given below, concentrating on those aspects that differ from, or are additional to BrainSignals. Full details of the piglet model can be found on the model website [34].

**Table 2.** Table of new model parameters.

parameter	value	source
[ADP]_n_	0.012 mM	[19]
[PCr]_n_	2.6 mM	[20]
[ATP]_n_	2.2 mM	[20]
[Py]_n_	0.1 mM	[1]
[gluc]_n_	1.2 mM	[21]
[gluc_c_]	5.3 mM	[15]
[lac]_n_	3.0 mM	[22]
[lac_c_]	2.0 mM	[15]
[PCr]_n_/[P_i_]_n_	1.5	[14,23]
*n*_a_	4.33	[24]
*k*_*m*,ATP_	0.025	[25]
*k*_PCr_	111 mM^−1^ s^−1^	^a^
*K*_eq, PCr_^*^	166	[26]
CMR_gluc,n_	25 µmol min^−1^100g^−1^	[27]
*k*_glut_	6.2 mM	[28]
*k*_MCT_	2.0 mM	[21]
*P*_vs_	1.5 mmHg	[29]
G_VArat,n_	4	[30]
*k*_*m*,glycG_	0.05 mM	[31]
*k*_*m*,glycA_	0.2 × [ADP]_n_	[6]
*k*_*m*,glycP_	0.2 × [P]_n_	[6]
*I*	3	[6]
*C*_v_	0.047 mmHg^−1^	[6]
*k*_pl_^−^	10 s^−1^	^a^
*k*_AK_	1055 s^−1^ mM^−1^	[6,25]
*k*_AK_^−^	379 s^−1^ mM^−1^	[6,25]
*k*_*m*,tcaP_	0.005 × [Py]_n_	[32]
*k*_*m*,tcaN_	0.6 × [NAD]_n_	[32]
*ΔG*°	−30.5 kJ mol^−1^	[33]

^a^Rate constants set to give fast reactions compared with other changes.

### 2.1. Circulation

The circulatory part of the model comprises three compartments: arteries and aterioles, capillaries and veins. The arterial and venous compartments have variable volumes *V*_a_ and *V*_v_, normalized such that the sum of their normal values (*V*_a,n_ and *V*_v,n_) is 1. For simplicity, the volume of the capillaries is ignored, as it is assumed to be small [35]. The arterial/arteriolar compartment has a variable conductance *G* which is sensitive to four inputs: the capillary oxygen concentration [O_2,c_], the mean arterial blood pressure *P*_a_, the arterial pressure of carbon dioxide *P*_a_CO_2_, and a parameter representing neuronal activation. The oxygen concentration affects conductance via the following equations.2.1
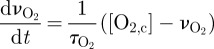
and2.2
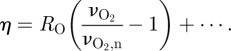


Here, 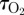 is a time constant and 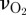 represents a low-pass filtered version of [O_2,c_] with normal value 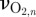. *R*_O_ is a parameter controlling the magnitude of the response. The muscular tension in the arterial wall *T*_max_ depends on *η* (which is the sum of the O_2_ term shown and three similar terms for *P*_a_CO_2_, arterial blood pressure and the demand parameter) in the following way:2.3
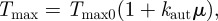
where *T*_max0_ is a constant, *k*_aut_ is a parameter representing the level of autoregulation, and *μ* is a sigmoidal function of *η*. An average vessel radius is calculated from the balance of pressures and tensions in the vessel wall. This in turn determines the conductance of the arterial/arteriolar tree via Poiseuille's law. A decrease in oxygen concentration therefore leads to an increase in the vessel radius and consequently an increase in blood volume and blood flow.

The venous compartment has a fixed resistance *G*_v_. The pressure at its boundary with the arteries *P*_v_ is calculated by equating the cerebral blood flow (CBF) through the arteries and veins giving2.4
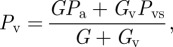
where *P*_vs_ is the pressure in the venous sinuses. The normalized venous volume *V*_v_ is then calculated by2.5
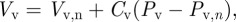
where *C*_v_ is the venous compliance, including a normalizing factor.

All blood compartments have a fixed haemoglobin concentration [Hb_tot_]. In each compartment, a fraction of this haemoglobin is oxygenated. These fractions are determined from the arterial oxygen saturation (a model input) and from the rate of oxygen transport. Dissolved oxygen from the capillaries diffuses directly to the mitochondrial compartment. The simulated NIRS haemoglobin signals *Δ*HHb and *Δ*HbO_2_ are calculated from the concentrations of oxy and deoxyhaemoglobin in the arteries and veins as follows:2.6
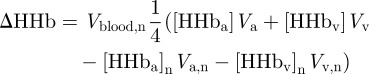
and2.7
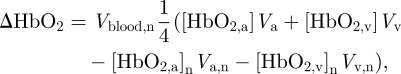
where [HHb] and [HbO_2_] are the concentrations of deoxy- and oxyhaemoglobin in the blood, and subscripts a and v refer to the arterial and venous compartments.

### 2.2. Metabolism

The metabolic part of BrainSignals is limited to oxidative phosphorylation. Particular attention is given to the CCO complex. This part of BrainSignals has been preserved, and extended to include ATP use and production, glycolysis and lactate dynamics. Many of the additional processes take place in the cytoplasm. However, with the exception of protons, no transport processes between cytoplasm and mitochondria are explicitly modelled. All other metabolites are considered in only one of these compartments.

**Figure 2.**
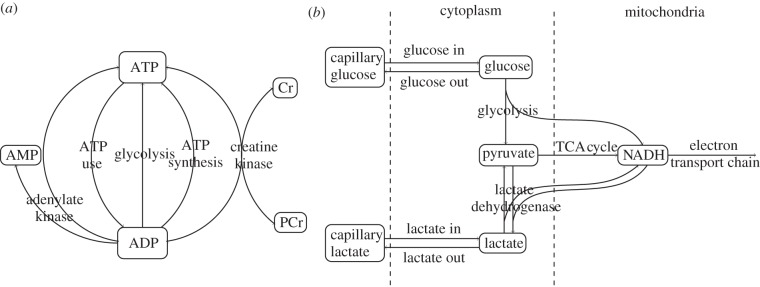
Diagrams of sections of the model that have been added to simulate MRS measured variables: (*a*) processes relating to ATP and (*b*) process relating to glycolysis, lactate and the TCA cycle.

An overview of the processes in the model involving the adenosine phosphates is shown in figure 2*a*. ATP is produced by ATP synthase in the mitochondria and by glycolysis in the cytoplasm. The rate of flow of protons through ATP synthase depends on the electrochemical potential *Δp* and the Gibbs free energy of ATP hydrolysis *ΔG* as follows:2.8
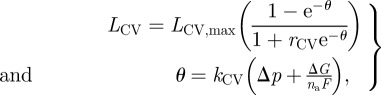
where *L*_CV,max_ is the maximum rate of proton flow, *r*_CV_ is a parameter controlling the relative forward and backward rates, and *F* is the Faraday constant. This is altered from BrainSignals to give a direct dependence on the energy state. The parameter *n*_a_ is the number of protons required to phosphorylate one ATP, and includes the proton used in the exchange of ADP and ATP across the mitochondrial membrane. The Gibbs free energy of ATP hydrolysis *ΔG* is calculated by2.9
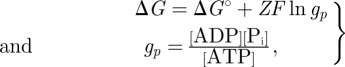
where *ΔG*° is the standard Gibbs free energy of ATP hydrolysis, *Z* is a thermodynamic constant, and the ratio *g*_*p*_ is known as the phosphorylation potential. All concentrations are taken to be cytoplasmic concentrations. The ATP synthesis rate is equal to *L*_CV_/*n*_a_.

ATP use is modelled as a single Michaelis–Menten process with a half-maximum concentration (*k*_*m*,ATP_) much lower than the normal ATP concentration. The reaction rate is therefore close to its maximum at normal ATP concentrations, and does not begin to decline significantly until ATP is low.

The final two processes influencing ATP concentration are buffering by phosphocreatine and AMP which occur with the following rates.2.10
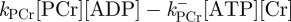
and2.11
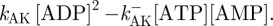


Both are modelled as mass action reactions and are assumed to reach equilibrium quickly. The ratio of forward and backward rate constants *k*_PCr_ and *k*_PCr_^−^ is equal to the effective equilibrium constant for the reaction *K*_eq,PCr_^*^.

The remaining metabolic processes relate to glycolysis and lactate dynamics, and link these to the electron transport chain via the production of nicotinamide adenine dinucleotide (NAD) as shown in figure 2*b*. The net rate of glucose transport is2.12
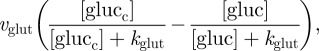
where [gluc] is the glucose concentration in the cytoplasm and [gluc_c_] is the glucose concentration in the capillary, which is fixed. The maximum rate *v*_glut_ is set so that when glucose concentration is at its normal value [gluc]_n_, the net transport rate is equal to the normal metabolic rate of glucose consumption CMR_gluc,n_. The same form is used for lactate transport, with the net transport rate given by2.13
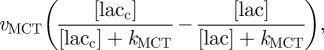
where [lac] is the lactate concentration in the cytoplasm, and [lac_c_] is the capillary lactate concentration which is fixed. Transport of lactate between brain cells has been the subject of much recent modelling, owing to the interest in the neuron–lactate aspartate shuttle [36–38]. Neurons and astrocytes have different expression of the MCT family members, leading to different *k*_MCT_ [39]. In our model however, there is no distinction between the two cell types, and we use an intermediate value for *k*_MCT_. The parameter *v*_MCT_ is calculated from the normal cerebral metabolic rate of lactate consumption CMR_lac,n_. If the model is to be at a steady state under normal conditions, the stoichiometry of the reactions gives an expression for CMR_lac,n_
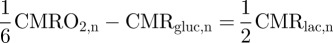


Satisfying this equation with the chosen values for CMR_gluc_ and CMRO_2_ results in a net production of lactate at baseline conditions.

Glycolysis is modelled as a single-stage Michaelis–Menten process with a rate dependent on glucose, ADP and P_i_ concentration. The maximum rate *v*_glyc_ is given by2.14
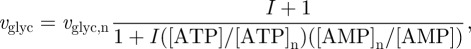
where *v*_glyc,n_ is the normal maximum rate of glycolysis. This expression represents the regulation by ATP and AMP of phosphofructokinase, an enzyme that catalyses an important regulatory step in glycolysis [40]. The parameter *I* controls how much the rate of glycolysis can change for given changes in ATP and AMP concentrations.

The interconversion between pyruvate and lactate is modelled as a mass action reaction with rate2.15
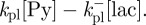


This reaction involves a proton, and the rate constants are related by2.16
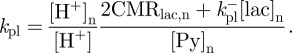


The magnitude of the rate constants is set to give a ratio that adapts effectively instantly to pH changes. This reaction, and glycolysis, also involve NAD, but NAD in the cytoplasm is not modelled. However, the reactions cause a conversion between mitochondrial NAD and the reduced form of NAD (NADH) to give the correct stoichiometry.

The transformation of pyruvate to acetyl-CoA and the whole tricarboxylic acid (TCA) cycle are combined into a single reaction that consumes one molecule of pyruvate and produces five reducing equivalents. These are all assumed to be NADH for simplicity. The rate is given by2.17
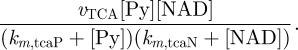


The *k*_*m*_ values were estimated from the detailed model of the TCA cycle by Wu *et al.* [32]. The *k*_*m*_ for pyruvate is small, since this model suggests that TCA cycle rate is not sensitive to pyruvate concentration unless it falls very low. The rate of the TCA cycle is also influenced by ATP and ADP concentrations. However, the main controlling factor is thought to be the NAD/NADH ratio [41].

The electron transport chain is described by three reactions. The first is the transfer of four electrons from NADH to the Cu_A_ centre of CCO. The second is the transfer of electrons from here to the cytochrome a_3_ centre, and the final reaction is the reduction of oxygen to water. The rates are calculated based on thermodynamic principles. The rate of the third reaction, which is proportional to CMRO_2_, is given by2.18
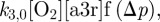
where *k*_3,0_ is a constant, *f* a function of the proton motive force *Δp*, [O_2_] the mitochondrial oxygen concentration and [a3r] the concentration of reduced cytochrome a_3_.

### 2.3. Implementation

Model development, simulations and analysis were carried out using the BRAINCIRC modelling environment [42]. Steady-state simulations were used to analyse the model behaviour, and dynamic simulations were used to compare the model with experimental data.

## 3. Results

**Figure 3.**
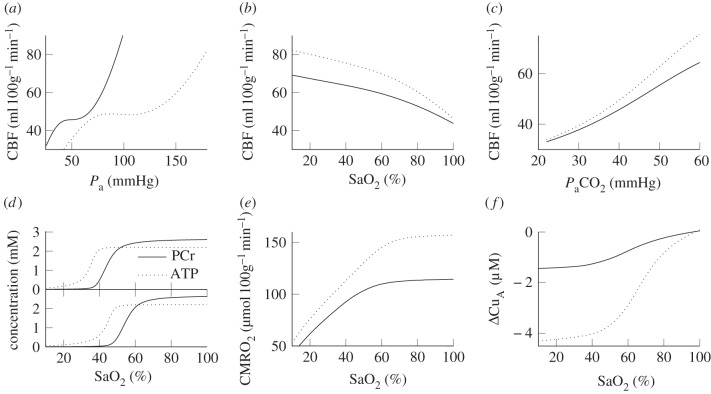
Steady-state simulations. (*a*–*c*) CBF versus arterial blood pressure, arterial oxygen saturation and arterial CO_2_ pressure. Solid lines were simulated with the piglet parameter values and dotted lines with the adult values shown in table 1. (*d*) PCr and ATP concentration versus arterial oxygen saturation for (top) piglet parameter values and (bottom) adult parameter values. (*e*) and (*f*) CMRO_2_ and change in Cu_A_ redox state versus arterial oxygen saturation for piglet parameter values (solid) and adult parameter values (dotted).

The steady-state predictions by the model of CBF with changes in blood pressure, oxygen saturation and arterial carbon dioxide tension (*P*_a_CO_2_) are shown in figure 3. The changes in PCr and ATP concentration, CMRO_2_ and *Δ*Cu_A_ with arterial oxygen saturation are also shown. These simulations are compared with the equivalent simulations with the parameter values for adults shown in table 1.

### 3.1. Simulation of anoxia

The model was used to simulate a previous experiment involving brief anoxias in six newborn piglets [12]. The piglets were anaesthetized with isoflurane and artificially ventilated. Their inspired oxygen was reduced from 40 per cent to 0 for 105 s. After returning to 40 per cent O_2_ for 10 min, the anoxia was repeated six times for each piglet. Mean arterial blood pressure (MAP) was monitored throughout, and continuous NIRS and ^31^P MRS measurements were recorded. The results showed a decrease in *Δ*HbO_2_ and increase in *Δ*HHb during anoxia. Immediately after anoxia there was an increase in total haemoglobin concentration indicating an increase in cerebral blood volume. There was a reduction of Cu_A_ during anoxia and a small hyperoxidation upon reoxygenation. The MRS results showed a decrease in PCr and inorganic phosphate concentration, but no changes in ATP concentration were seen.

**Figure 4.**
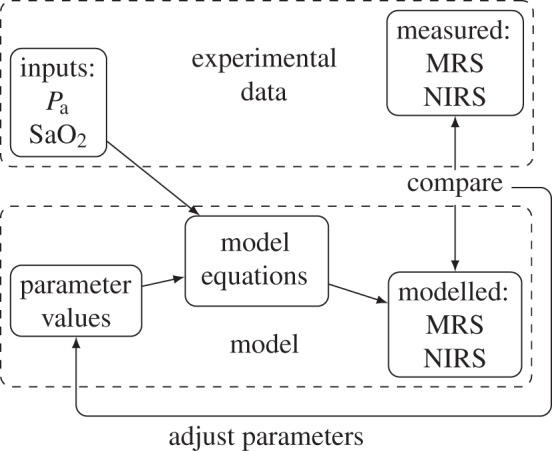
Flow chart showing the modelling process.

The MAP and arterial oxygen saturation (SaO_2_) were used as inputs to the model, and its outputs were compared with the averaged NIRS and MRS measurements, as illustrated in figure 4.

**Figure 5.**
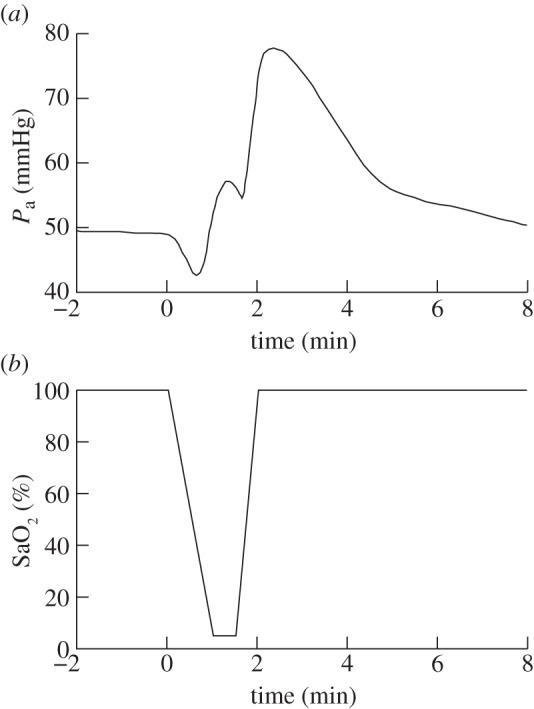
(*a*) Arterial blood pressure and (*b*) arterial oxygen saturation used as model inputs to simulate anoxia [12]. Blood pressure was taken from the measurements from one piglet. SaO_2_ was estimated from the experimental protocol.

**Figure 6.**
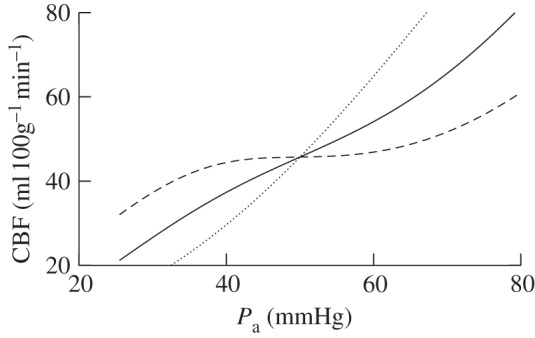
Steady-state CBF versus mean arterial blood pressure after the parameter changes shown in table 3 but with *k*_aut_ = 1 (dashed line) 0.5 (solid line) and 0 (dotted line). CBF changes (in the range 30–70 mmHg) are 0.8, 2.1 and 3.8%/mmHg, respectively.

**Figure 7.**
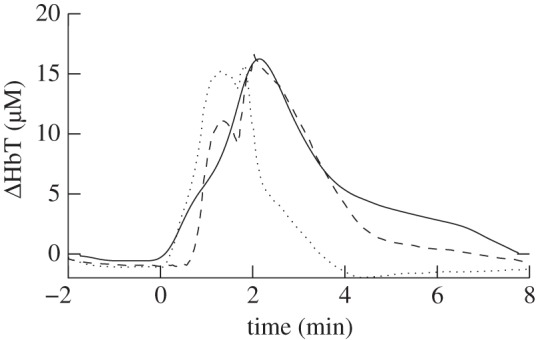
Measured (solid line) total change in haemoglobin concentration (*Δ*HHb + *Δ*HbO_2_) and simulated with *k*_aut_ = 0.5 (dashed line) and *k*_aut_ = 1.0 (dotted line) with the parameter value changes shown in table 3.

Carbon dioxide levels were not reported during the challenge and assumed to remain constant. Oxygen saturation measurements were not available, so SaO_2_ was estimated from the experimental protocol. This estimate is shown, along with the measured MBP, in figure 5. Comparisons between modelled and measured results showed some significant differences. By changing four parameters that were known to affect relevant parts of the model, the simulations were improved. These changes are listed in table 3 and are explained as follows:

—the autoregulation capacity in the model was decreased by reducing the parameter *k*_aut_ from 1.0 to 0.5. After this change, the maximum venous volume increase was approximately 45 per cent. The effect of this change on the autoregulation curve is shown in figure 6. The change in CBF as a percentage of baseline between 30 and 70 mmHg increased from 0.8% mmHg^−1^ with *k*_aut_ = 1.0 to 2.1% mmHg^−1^ with *k*_aut_ = 0.5. The improvement in the simulation of total haemoglobin concentration changes is shown in figure 7;
—the glycolysis rate was made more sensitive to changes in AMP and ATP concentration by increasing the parameter *I* from 3 to 50. This resulted in an approximately sevenfold increase in glycolysis rate during anoxia. Also, when *I* = 50, the PCr concentration at the end of anoxia was 55 per cent of its baseline value compared with 25% when *I* = 3;
—the normal ratio of PCr to P_i_ was increased from 1.5 to 2.7. In the model, if ATP concentration is constant, changes in PCr and P_i_ concentration are equal and opposite (ignoring AMP which is present only at very small concentrations). The experimental results therefore require a ratio of [PCr]_n_ to [P_i_]_n_ of 2.7. With this ratio, the model will accurately simulate the P_i_ measurements providing the PCr measurements are accurately simulated;
—the normal NAD/NADH ratio was decreased from 9 to 1.5. This resulted in a decrease in the rate of Cu_A_ reduction.

**Table 3.** Table of model parameters that were changed to improve the model simulations of anoxia.

parameter	default value	changed value
[PCr]_n_/[P_i_]_n_	1.5	2.7
*I*	3	50
*k*_aut_	1.0	0.5
[NAD]_n_/[NADH]_n_	9.0	1.5

**Figure 8.**
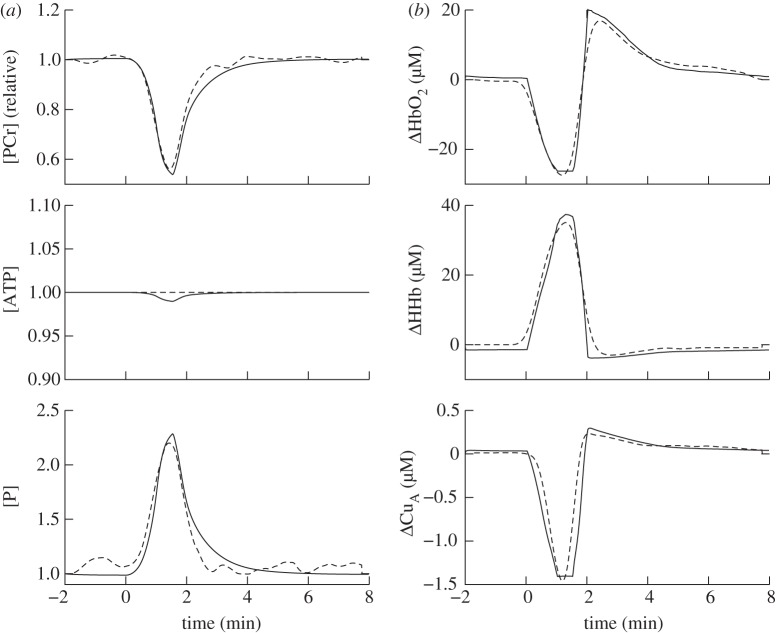
Model simulations (solid line) compared with measurements (dashed line) from NIRS (*b*) and MRS (*a*) during anoxia in piglets, taken from Springett *et al.* [12]. Parameter values for the model were set to those in table 3.

**Figure 9.**
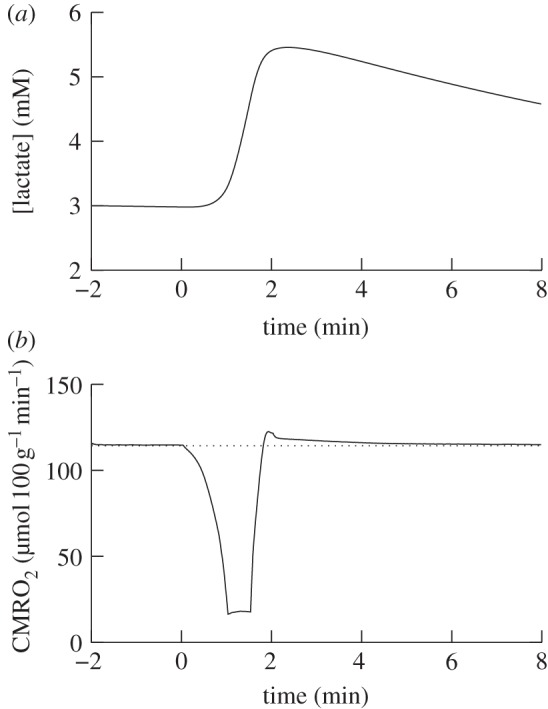
(*a*) Model predictions of lactate concentration and (*b*) CMRO_2_ with normal CMRO_2_ shown as dotted during anoxia.

The modelled and measured results after implementing these changes are shown in figure 8. The model's predictions for lactate concentration and CMRO_2_ are shown in figure 9. CMRO_2_ was predicted to decrease to 15% of its baseline value during anoxia and increase to 108% during reoxygenation.

**Figure 10.**
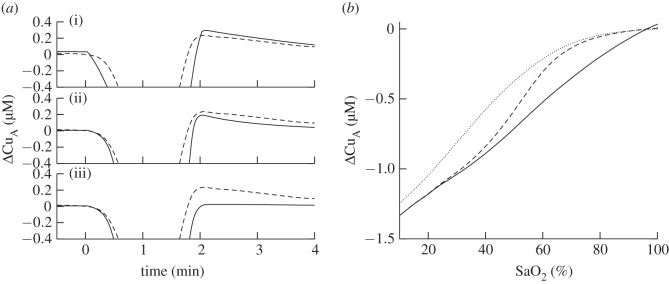
(*a*) Comparison of measured Cu_A_ signal (dashed) with modelled Cu_A_ signal (solid) for (from top to bottom) (i). The model with its default equations, and parameter values from table 3 (ii). As (i), but with an altered CMRO_2_ dependence on oxygen (equation (2.18) replaced with equation (3.1)) (iii). As (ii), but with no dependence on phosphorylation potential of ATP synthesis rate (*Δ*G replaced with its normal value in equation (2.8)). (*b*) *Δ*Cu_A_ vs oxygen saturation at the onset of anoxia for measured data (dashed) model as in (i) on left (solid) and model as in (ii) on left (dotted).

In addition to these changes, the model was altered to explore the simulation of the Cu_A_ redox state. This was done to investigate the hyperoxidation seen following anoxia, which is seen in the data but is not well understood. Initially, equation (2.18) was changed to3.1
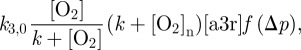
where *k* = 5 µM. The model was then further changed such that there was no dependence of ATP synthesis rate on phosphorylation potential by replacing *ΔG* with its normal value in equation (2.8). The results of these changes are shown in figure 10. When equation (3.1) is used, the simulation of *Δ*Cu_A_ is significantly improved as SaO_2_ begins to drop (although the simulation is slightly worse at lower SaO_2_). Also, the magnitude of hyperoxidation of Cu_A_ during recovery is decreased. When, in addition, the dependence of ATP synthesis rate on phosphorylation potential is removed, no hyperoxidation is seen.

## 4. Discussion

The model has been applied successfully to simulate oxygenation and metabolic changes in the brains of newborn piglets during anoxia. It correctly predicted no significant changes in ATP concentration throughout the experiment. To simulate the other measured signals, changes to the original model were required. These changes offer an insight into the physiological interpretation of the experimental results as discussed below. In addition, the model allows predictions of variables that are more difficult to measure, including CMRO_2_ and lactate concentration.

During recovery from anoxia, a large increase in MAP accompanied by an increase in [HbO_2_] was measured. As there was not a corresponding reduction in [HHb], this implies an increase in cerebral blood volume. The model suggests that this hyperaemia cannot be attributed to changes in the arterial volume alone. A dilation of the cerebral arteries to this extent would be predicted to cause a several-fold increase in CBF, which, unless accompanied by a corresponding increase in CMRO_2_, would lead to a decrease in [HHb] via a washout effect. It is more likely, therefore, that the increased blood volume following anoxia has a significant venous contribution, which is driven by the increase in blood pressure. However, the autoregulation behaviour of the model causes changes in arterial pressure of this magnitude to be damped by changes in arterial resistance before they are felt at the veins. A reduction of the parameter *k*_aut_ from 1.0 to 0.5 reduced this cancelling effect, and so led to a better replication of the observed hyperaemia. Previous studies in piglets have found that CBF does not change in the range of 50–80 mmHg [43–45]. However, these studies used piglets older than 24 h. In one study involving piglets of different ages, it was found that for piglets less than 4 days old, the average change in CBF as blood pressure was varied was 1.1%/mmHg, whereas for piglets older than 4 days old it was 0%/mmHg [46]. The interpretation of the anoxia measurements using our model suggests that cerebral autoregulation was impaired or not fully developed in these newborn piglets.

The relationship between PCr and ATP concentration in the model as oxygen saturation falls shows the expected buffering of ATP by PCr, with ATP not decreasing until PCr is low. The model predicts that ATP concentration will begin to decrease when SaO_2_ falls below 40 per cent. However, the model predicted a drop in PCr concentration during anoxia larger than that observed. Increasing the parameter *I* allowed a greater increase in glycolysis rate, and therefore a greater rate of ATP synthesis during anoxia. This glycolysis rate could not be sustained for longer anoxias, because the model predicts that glucose is used faster than its maximum rate of transport into the cell. The value of *I* was increased from 3 to 50. Above this value, there is almost no further change in glycolysis rate, because changes in ATP and AMP concentrations become limiting. For large *I*, the glycolysis rate in the model increased approximately sevenfold. This is greater than the fivefold increase calculated to occur in foetal rats during ischaemia [47]. It should be noted that the extent of PCr concentration decrease in the model is also sensitive to the normal PCr concentration [PCr]_n_. There are two features of particular interest in the Cu_A_ experimental results: the delay between the decrease in HbO_2_, and the reduction of Cu_A_, and the hyperoxidation of Cu_A_ during recovery. The former is not reproduced by the model in its original form, but the latter is. It has been observed previously in piglets that Cu_A_ redox state is not affected by mild hypoxia [48] i.e. CCO has a low apparent *k*_*m*_ for oxygen. A similar effect has also been observed in adult rats [49,50], but not in adult humans [51,52]. The mechanisms for this effect are unknown, and it does not emerge from our alterations of the BrainSignals model to simulate the neonatal piglet brain. But, when included directly in the model, it improved the simulation of Cu_A_ reduction at the onset of anoxia. As expected, it also decreased the magnitude of Cu_A_ hyperoxidation following anoxia, since this is partly caused by the increased oxygen tension at the mitochondria as a consequence of the increased oxygen delivery. An oxidation is still seen however, because the increased concentrations of ADP and P_i_ during and following anoxia increase the rate of ATP synthesis via equations (2.8) and (2.9). The proton motive force across the mitochondrial membrane is decreased which increases CMRO_2_ and, with the parameter values used, results in an oxidation of Cu_A_. If this dependency on phosphorylation potential is removed, no oxidation is seen. This supports the suggestion of Springett *et al.* [12] when reporting the experimental results, that the hyperoxidation was a result of an increased CMRO_2_. A Cu_A_ hyperoxidation following anoxia was also observed in an earlier study [53]. The authors suggest that a pH drop could partly be responsible for the oxidation via a direct affect of pH on Cu_A_. There is no mechanism for this effect in our model, although a small change in pH was observed following anoxia, as measured by MRS.

Another possible explanation for the oxidation is a decrease in the substrate supply to the TCA cycle. Our model suggests the opposite is true for pyruvate, whose concentration increases during anoxia, and takes several minutes to return to normal. However, there is an increase in NADH/NAD ratio which will tend to decrease the rate of the TCA cycle; but this appears to have only a small effect on the oxidation of Cu_A_. The NAD/NADH ratio does however have a significant effect on the rate of reduction of Cu_A_. Although changes in mitochondrial NADH concentration can be measured by fluorescence, the NAD/NADH ratio is difficult to measure *in vivo* [54]. Estimates of this ratio differ, for example in rat liver cells a ratio of 5–10 was measured [55], but in myocytes isolated from newborn piglets a ratio of 1.2 was found [56]. We found a decrease in the model's normal NAD/NADH ratio from 9 to 1.5 led to the simulated rate of Cu_A_ reduction matching the experimental rate. However, there are other factors that affect this rate, including the form of the TCA cycle rate dependence on NAD/NADH ratio, and the sensitivity of the reactions of the electron transport chain to changes in the proton gradient. This part of our model requires further investigation.

There are some limitations of our model. In general, we wish to keep the model as simple as possible, while still accurately replicating experimental results in a way that is representative of the underlying physiology. We believe that this allows a greater understanding of the model's behaviour. However, some aspects may have been oversimplified. In particular, it may be necessary to model NAD, NADH and pH in the cytoplasm. The ratio of NAD to NADH in the cytoplasm is much larger than in the mitochondria [55]. Changes in these variables affect the lactate to pyruvate ratio, and the rate of glycolysis. Also, we have not distinguished between astrocytes and neurons. Their different functions are important when considering lactate dynamics. Finally, for the anoxia experiment simulated here, the assumption of constant arterial CO_2_ concentration is unlikely to be correct. CO_2_ changes have an important effect on blood flow, and may have affected the simulation of the NIRS haemoglobin signals.

We have used a physiological approach to construct our model. The main strength of this type of modelling is that it allows hypotheses to be generated and tested. However, it can lead to more complex models, and this makes it more difficult to analyse. Therefore, some components that have been simplified are treated phenomenologically. In this paper, we relied on knowledge of the model and experimentation to identify parameters with important effects. In the future, we will implement a more rigorous sensitivity analysis to analyse the effects of all parameters on particular model outputs. We are currently using the model to simulate MRS and NIRS measurements during HI in piglets. These measurements have complex relationships with one another, and we will use the model to help understand them in the context of our biological knowledge. We will also use it to simulate new treatments such as xenon-augmented hypothermia which is being investigated in our group [57].
